# Eosinophils as immunoregulatory players in allergic rhinitis: beyond cytotoxicity

**DOI:** 10.3389/fimmu.2026.1888525

**Published:** 2026-07-08

**Authors:** Xinyu Han, Fei Li, Feihu Wu, Chunsong Liu, Qiqi Yang, Yan Ruan, Weiping He, Wenda Feng, Gang Liu, Chunqiao Li

**Affiliations:** 1 The First Affiliated Hospital of Guangzhou University of Chinese Medicine, Guangzhou, Guangdong, China; 2 ShenShan Hospital, The First Affiliated Hospital of Guangzhou University of Chinese Medicine, Shanwei, Guangdong, China; 3 Second Affiliated Hospital of Anhui University of Chinese Medicine, Hefei, Anhui, China; 4 Department of Otorhinolaryngology-Head and Neck Surgery, The First Affiliated Hospital of Anhui University of Chinese Medicine, Hefei, Anhui, China; 5 Department of Otolaryngology Head and Neck Surgery, The First Affiliated Hospital of Guangzhou University of Chinese Medicine, Guangzhou, Guangdong, China; 6 Lingnan Institute of Otolaryngology, Guangdong Clinical Research Academy of Chinese Medicine, Guangzhou, Guangdong, China

**Keywords:** allergen immunotherapy, allergic rhinitis, eosinophil, eosinophil heterogeneity, IL-10, immunoregulation, specialized pro-resolving mediators, TGF-β

## Abstract

Eosinophils have traditionally been viewed as terminal effector cells in allergic rhinitis (AR), driving tissue damage through the release of cytotoxic granule proteins and pro-inflammatory mediators. However, accumulating evidence over the past two decades has challenged this paradigm, revealing that eosinophils harbor a substantial immunoregulatory repertoire. This Mini Review synthesizes emerging findings on four underappreciated dimensions of eosinophil immunoregulatory function in the context of AR: (i) the capacity of eosinophils to produce interleukin-10 (IL-10) and engage in functional crosstalk with Foxp3+ regulatory T cells (Tregs); (ii) the dual role of eosinophil-derived transforming growth factor-beta (TGF-β) in both tissue remodeling and mucosal tolerance; (iii) eosinophil lipid mediator class-switching from pro-inflammatory cysteinyl leukotrienes (CysLTs) to specialized pro-resolving mediators (SPMs); and (iv) the functional heterogeneity between tissue-resident and inflammatory eosinophil subsets. We further discuss the clinical implications of these findings, including the paradoxical outcomes of anti-IL-5 therapies in AR and the potential of immunoregulatory eosinophil markers as biomarkers for allergen-specific immunotherapy (AIT). A deeper understanding of the context-dependent immunoregulatory functions of eosinophils in the nasal mucosa may inform precision therapeutic strategies that selectively target pro-inflammatory eosinophil subsets while preserving their homeostatic and tolerogenic roles.

## Graphical Abstract

**Figure:**
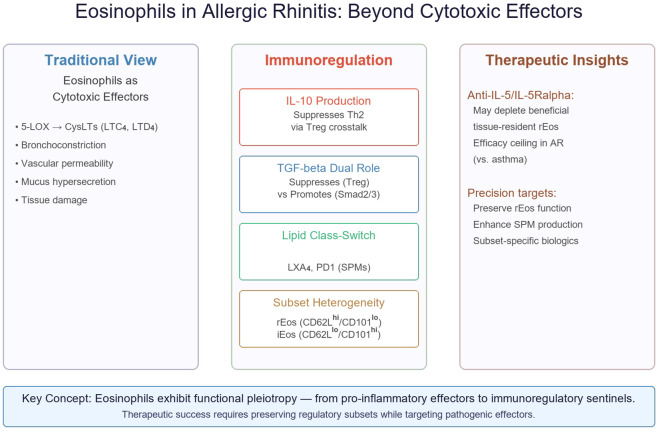
Overview of eosinophil functional pleiotropy in allergic rhinitis. Schematic overview of eosinophil functional pleiotropy in allergic rhinitis (AR). Left panel: traditional pro-inflammatory pathways — eosinophils produce cysteinyl leukotrienes (CysLTs: LTC_4_, LTD_4_) via 5-lipoxygenase (5-LOX)/FLAP, driving bronchoconstriction, vascular permeability, mucus hypersecretion, and tissue damage. Center panel: four emerging immunoregulatory dimensions (1). IL-10/Treg axis: eosinophils produce IL-10, which promotes the expansion of Foxp3+ regulatory T cells (Tregs) in an MHC-II-dependent manner; AIT enhances IL-10 mRNA expression in nasal mucosal eosinophils (2). TGF-β dual role: eosinophil-derived TGF-β1 (~25 pg/cell reservoir) exerts context-dependent effects — at sustained high concentrations it promotes tissue fibrosis via collagen deposition and TIMP-1 upregulation, whereas at lower concentrations it facilitates mucosal tolerance through Treg induction and IgA class-switching (3). Lipid mediator class-switching: eosinophils co-express 5-lipoxygenase (5-LOX)/FLAP and 15-lipoxygenase-1 (15-LOX-1), endowing them with the capacity to switch lipid mediator biosynthesis from pro-inflammatory cysteinyl leukotrienes (CysLTs: LTC_4_, LTD_4_) to specialized pro-resolving mediators (SPMs: LXA_4_, PD1) under permissive microenvironmental conditions (4). Functional heterogeneity: eosinophils comprise two functionally distinct subsets — CD62L^hi^/CD101^lo^ tissue-resident eosinophils (rEos) that mediate immune homeostasis, and CD62L^lo^/CD101^hi^ inflammatory eosinophils (iEos) that execute classical pro-inflammatory effector functions. The differential sensitivity of rEos versus iEos to anti-IL-5 therapies may partly explain the partial clinical efficacy of biologics in AR. Right panel: therapeutic implications — anti-IL-5/IL-5Rα biologics may inadvertently deplete beneficial tissue-resident rEos, partially explaining the efficacy ceiling in AR; precision strategies should preserve rEos function, enhance SPM production, and target subset-specific pathways. Abbreviations: AIT, allergen-specific immunotherapy; CysLTs, cysteinyl leukotrienes; EOS, eosinophil; FLAP, 5-LOX-activating protein; 5-LOX, 5-lipoxygenase; 15-LOX-1, 15-lipoxygenase-1; MHC-II, major histocompatibility complex class II; rEos, tissue-resident eosinophils; iEos, inflammatory eosinophils; SPMs, specialized pro-resolving mediators; TIMP-1, tissue inhibitor of metalloproteinases-1.

## 1 Introduction

Eosinophils were first described by Paul Ehrlich in 1879, and for over a century they have been predominantly characterized as terminal effector cells in type 2 inflammatory responses, particularly in allergic diseases and helminth infections (1). In allergic rhinitis (AR), tissue eosinophilia is strongly correlated with symptom severity, and eosinophil-derived products — including eosinophil cationic protein (ECP), major basic protein (MBP), and eosinophil peroxidase (EPO) — have been consistently detected in nasal lavage fluid following allergen challenge (2, 3). This canonical view has positioned eosinophils as unequivocal drivers of mucosal inflammation and tissue injury.

Yet a growing body of evidence has begun to dismantle this unidimensional framework. Fundamental insights have emerged from eosinophil-deficient mouse models (ΔdblGATA and PHIL mice), which unexpectedly revealed that eosinophils contribute to non-immunological homeostatic processes, including mammary gland development, metabolic regulation in adipose tissue, and maintenance of plasma cells in the bone marrow (4–6). These findings catalyzed a conceptual shift: eosinophils are not merely tissue-damaging effector cells but orchestrate immunoregulatory functions through cytokine secretion, lipid mediator biosynthesis, and direct cell–cell interactions (7, 8).

Several observations in AR further underscore this paradox. Subclinical sensitization — detectable in asymptomatic individuals through skin prick testing — suggests that the presence of allergic sensitization per se does not necessarily equate to clinical disease, and that additional local factors are required for the development of clinical symptoms (9). Moreover, successful AIT is associated with an increase rather than a decrease in local eosinophil counts during the early treatment phase, which is counterintuitive under the classical cytotoxicity model (10, 11). These clinical puzzles compel a reconceptualization of eosinophil function beyond the canonical effector paradigm.

In this Mini Review, we focus specifically on the immunoregulatory dimensions of eosinophils as they pertain to AR, an area that has received far less attention compared to the extensive literature on eosinophil regulation in asthma and eosinophilic esophagitis. We synthesize current evidence across four emerging themes — IL-10 production, TGF-β-mediated dual signaling, lipid mediator class-switching, and subset heterogeneity — and discuss their implications for therapeutic strategies and biomarker development in the management of AR.

## 2 Emerging immunoregulatory functions of eosinophils in allergic rhinitis

### 2.1 IL-10-producing eosinophils and Treg crosstalk

Interleukin-10 (IL-10) is a canonical immunoregulatory cytokine that suppresses T-cell proliferation, inhibits pro-inflammatory cytokine production, and promotes the differentiation of Foxp3+ regulatory T cells (Tregs) (12). While Tregs and regulatory B cells are the most extensively studied sources of IL-10 in the context of allergy, eosinophils have also been identified as a constitutive and inducible source of this cytokine.

The capacity of eosinophils to produce IL-10 was first systematically demonstrated by Lotfi et al. (13), who showed that human peripheral blood eosinophils constitutively express IL-10 mRNA and protein, and that IL-5 stimulation further upregulates IL-10 production (13). Subsequent studies have further characterized the IL-10-producing capacity of eosinophils, demonstrating that eosinophils constitutively store multiple immunoregulatory cytokines including IL-10 (14), and that CD69 receptor engagement provides an activation signal for enhanced IL-10 production by tissue eosinophils (15). Functionally, IL-10+ eosinophils have been shown to promote the expansion of Foxp3+ Tregs *in vitro*, and this effect is partially dependent on eosinophil expression of major histocompatibility complex class II (MHC-II), which enables antigen presentation to naive CD4+ T cells (16, 17).

Within the context of AR, a particularly intriguing observation is the temporal dynamics of IL-10+ eosinophils during AIT. Nouri-Aria et al. (18) demonstrated that successful grass pollen immunotherapy was associated with a significant increase in IL-10 mRNA-expressing cells in the nasal mucosa, with eosinophils identified as a prominent cellular source alongside T cells (18). Shamji et al. (19) corroborated these findings, showing that functional suppression of allergen-stimulated responses during AIT correlated with increased IL-10 production by multiple cell types, including eosinophils (19). These longitudinal clinical data suggest that eosinophils may participate actively — rather than passively — in the establishment of allergen-specific tolerance during AIT.

This perspective challenges the long-held assumption that AIT works by simply eliminating or depleting eosinophils from the nasal mucosa. Instead, AIT may functionally reprogram eosinophils from a pro-inflammatory to an immunoregulatory phenotype, with IL-10 serving as both a mediator and a marker of this reprogramming (20). Whether IL-10+ eosinophils in the nasal mucosa can serve as an early predictive biomarker for AIT clinical response remains an open question warranting prospective investigation.

### 2.2 TGF-β secretion by eosinophils and the nasal remodeling paradox

Transforming growth factor-beta (TGF-β) is a pleiotropic cytokine with context-dependent functions spanning fibrosis, immune tolerance, and tissue repair (21). Eosinophils were recognized as a prominent source of TGF-β by Levi-Schaffer and colleagues, who demonstrated that eosinophils store pre-formed TGF-β1 in their secretory granules and release it upon activation (22). Subsequent studies quantified that a single eosinophil can contain up to 25 pg of TGF-β1, positioning eosinophils as one of the most concentrated cellular reservoirs of this cytokine (23).

In allergic airway disease, eosinophil-derived TGF-β has traditionally been implicated in subepithelial fibrosis and airway remodeling, processes well characterized in asthma but far less pronounced in AR (24, 25). This discrepancy — the ‘nasal remodeling paradox’ — raises an important question: does eosinophil-derived TGF-β in the nasal mucosa serve a fundamentally different function than in the lower airways?

Accumulating evidence supports a dual role for eosinophil-derived TGF-β that depends on local concentration and co-stimulatory signals. At low concentrations, TGF-β promotes the differentiation of naive CD4+ T cells into Foxp3+ Tregs, thereby contributing to mucosal immune tolerance (26). At high and sustained concentrations, TGF-β synergizes with IL-4 and IL-13 to drive myofibroblast differentiation and collagen deposition, promoting fibrosis (27). In the nasal mucosa, where eosinophil burden is typically lower and episodic compared to the persistently inflamed asthmatic airway, TGF-β may tilt toward its immunoregulatory rather than fibrotic function. This hypothesis is supported by histological studies showing that the degree of subepithelial fibrosis in AR is substantially milder than in asthma, despite comparable eosinophil infiltration during exacerbations (28).

Furthermore, recent work by Gurtner et al. (29) demonstrated that eosinophil-derived TGF-β drives the expansion of peripherally induced neuropilin-negative RORγt+ regulatory T cells during bacterial and allergen challenge at mucosal surfaces, providing direct evidence of an eosinophil–Treg regulatory circuit mediated by TGF-β (29). While this study focused on the intestinal and pulmonary mucosa, the conceptual framework is directly applicable to the nasal mucosa and warrants tissue-specific investigation in AR.

### 2.3 Lipid mediator class-switching: from leukotrienes to pro-resolving mediators

Eosinophils are equipped with an elaborate enzymatic machinery for arachidonic acid metabolism, most notably the 5-lipoxygenase (5-LOX)/5-LO-activating protein (FLAP) pathway and LTC_4_ synthase, enabling them to produce large quantities of cysteinyl leukotrienes (CysLTs), including LTC_4_, LTD_4_, and LTE_4_ (30). CysLTs are potent bronchoconstrictors and pro-inflammatory mediators that enhance vascular permeability and mucus secretion, and their pathogenic role in AR is well established (31).

However, eosinophils also constitutively express 15-lipoxygenase-1 (15-LOX-1), an enzyme that initiates the biosynthesis of specialized pro-resolving mediators (SPMs), including lipoxins (e.g., LXA_4_) and protectins (e.g., PD1) (32, 33). Recent studies have provided direct evidence that eosinophils can actively generate SPMs: Miyata et al. (34) demonstrated that eosinophil-derived 15-LOX-1 contributes to the production of protectin D1 (PD1) and lipoxin A_4_ (LXA_4_), and Miyata et al. (34) further showed that eosinophils are a cellular source of SPMs in type 2 inflammatory conditions (35, 36). This dual enzymatic capacity — the co-expression of both 5-LOX and 15-LOX-1 — endows eosinophils with the ability to switch lipid mediator output from predominantly pro-inflammatory CysLTs to pro-resolving SPMs depending on microenvironmental cues.

The concept of ‘lipid mediator class-switching’ was articulated by Levy et al. (37), who proposed that during the natural resolution phase of acute inflammation, a shift in the available substrate composition and enzyme localization redirects arachidonic acid metabolism from the 5-LOX/CysLT axis toward the 15-LOX/SPM axis (37). Prostaglandin E_2_ (PGE_2_) may regulate this class-switching by promoting 15-LOX-1 expression while suppressing 5-LOX activity via the cAMP-PKA pathway (35, 38). Direct experimental evidence supporting PGE_2_-mediated regulation of the 5-LOX/15-LOX-1 balance in eosinophils would further strengthen this mechanistic model.

The relevance of eosinophil lipid mediator class-switching to AR has been underexplored. Nasal allergen challenge produces a biphasic inflammatory response: an early phase dominated by mast cell degranulation and histamine release, followed by a late phase (4–24 hours) characterized by eosinophil infiltration and CysLT production (39). The natural resolution of this late-phase response, which occurs over 24–72 hours, is likely an active process mediated in part by SPMs. Whether eosinophils within the nasal mucosa switch from CysLT to SPM production during this resolution phase — and whether defective class-switching contributes to persistent AR — remains unknown. The identification of lipid mediator profiles (CysLTs vs. SPMs) in nasal lavage fluid as biomarkers of AR resolution status represents a compelling translational opportunity (39, 40).

### 2.4 Eosinophil subset heterogeneity: the tissue-resident regulatory phenotype

One of the most transformative developments in eosinophil biology over the past decade is the recognition that eosinophils are not a homogeneous population but comprise functionally distinct subsets. Seminal work by Mesnil et al. (41) demonstrated that lung eosinophils can be divided into two major subpopulations based on surface marker expression: CD62L^hi^/CD101^lo^ tissue-resident eosinophils (rEos) that are present under homeostatic conditions and participate in immunoregulation, and CD62L^lo^/CD101^hi^ inflammatory eosinophils (iEos) that are recruited during allergic inflammation and possess classical cytotoxic effector functions (42). Transcriptomic analysis of Siglec-F^tt^ lung eosinophils further revealed a profile enriched for genes involved in lipid metabolism, antigen presentation, and immunoregulation in the resident subset, supporting the concept that rEos possess a distinct functional repertoire (41).

The functional dichotomy between rEos and iEos has profound implications for AR. If rEos constitutively populate the human nasal mucosa and contribute to local immune homeostasis — analogous to their role in the lung and intestinal lamina propria (41) — then therapeutic strategies aimed at global eosinophil depletion (e.g., anti-IL-5 or anti-IL-5Rα biologics) may inadvertently remove a beneficial regulatory cell population. This mechanistic concern raises the possibility that the modest and inconsistent efficacy of anti-IL-5/anti-IL-5Rα biologics observed in AR (43–45), in contrast to their established efficacy in severe eosinophilic asthma, may partly reflect the unintended removal of immunoregulatory tissue-resident eosinophils. Direct evidence in AR is currently limited, and further studies are needed to clarify whether this concept applies to the nasal mucosa.

To date, the rEos versus iEos paradigm has been studied primarily in murine models and in the context of asthma and eosinophilic gastrointestinal disorders. The phenotypic landscape of eosinophil subsets in the human nasal mucosa — including their relative abundance, transcriptomic signatures, and functional polarization in AR versus health — has not been systematically characterized. Single-cell RNA sequencing of nasal mucosal biopsies from AR patients at baseline, during allergen challenge, and during AIT would be particularly informative. Furthermore, surface markers that reliably distinguish immunoregulatory from pro-inflammatory eosinophils in human tissues are urgently needed to enable the development of subset-selective therapeutic strategies (46–48).

## 3 Discussion

The evidence synthesized in this Mini Review supports a model in which eosinophils in AR are not monolithic pro-inflammatory effectors but rather context-dependent immunoregulatory cells — a ‘Dr. Jekyll and Mr. Hyde’ duality (49) determined by the local cytokine milieu, allergen exposure phase, and eosinophil subset composition (**Table 1**). This paradigm shift has several clinically relevant implications.

**Table 1:** Key evidence on eosinophil immunoregulatory functions in allergic rhinitis.

Function	Key molecules	Evidence in AR	Clinical implication
IL-10 production	IL-10, MHC-II	IL-10+ Eos increase during AIT (18, 19)	Biomarker for AIT response
TGF-β secretion	TGF-β, Foxp3	Nasal remodeling milder than asthma (28)	Tissue-specific regulatory role
Lipid class-switching	5-LOX, 15-LOX-1, LXA_4_, PD1	Late-phase SPM production (32, 36, 38)	Resolution-promoting strategy
Subset heterogeneity	CD62L, Siglec-F, CD101	rEos vs iEos not yet profiled in AR	Subset-selective targeting
Treg crosstalk	IL-10, TGF-β, IDO	Eos-Treg co-localization (17, 18)	Sparing rEos in anti-IL-5 therapy

AIT, allergen-specific immunotherapy; AR, allergic rhinitis; CysLTs, cysteinyl leukotrienes; iEos, inflammatory eosinophils; rEos, tissue-resident eosinophils; SPMs, specialized pro-resolving mediators; Tregs, regulatory T cells.

First, it offers a mechanistic explanation for the modest and inconsistent clinical efficacy of anti-IL-5/anti-IL-5Rα biologics in AR. If a substantial fraction of nasal mucosal eosinophils consists of tissue-resident rEos that produce IL-10 and TGF-β and contribute to mucosal immune homeostasis, then their indiscriminate depletion may counteract the intended therapeutic benefit of eliminating pro-inflammatory iEos. This concept — ‘collateral immunoregulatory damage’ — has been proposed in the context of anti-eosinophil therapies in asthma (48) but has not been explicitly examined in AR.

Second, the observation that successful AIT is accompanied by a functional reprogramming of eosinophils toward an IL-10-producing phenotype suggests that biomarkers based on eosinophil immunoregulatory capacity — rather than eosinophil abundance — may improve the prediction and monitoring of AIT outcomes. Potential candidate biomarkers include the IL-10-to-ECP ratio in nasal lavage fluid, the lipid mediator profile (CysLTs vs. SPMs), and the rEos-to-iEos ratio in nasal cytology samples (47).

Third, the future development of subset-selective therapeutic approaches — agents that selectively deplete iEos while sparing rEos, or that pharmacologically promote lipid mediator class-switching toward SPM production — represents a conceptually attractive strategy that merits preclinical investigation.

Several open questions delineate the frontier of this field: (i) what is the relative proportion of rEos versus iEos in the healthy versus AR nasal mucosa, and how does this ratio change during AIT?; (ii) through what molecular mechanisms does AIT reprogram eosinophils from a pro-inflammatory to an immunoregulatory phenotype?; (iii) can the IL-10-producing capacity of nasal eosinophils be harnessed as an early predictive biomarker of AIT efficacy?; and (iv) does the clinical inefficacy of anti-IL-5 therapies in AR reflect the detrimental removal of immunoregulatory eosinophils, and can this be tested in clinical trials with tissue-level eosinophil subset profiling?

## 4 Conclusion

Eosinophils in allergic rhinitis harbor a substantial yet underrecognized immunoregulatory capacity that encompasses IL-10-mediated Treg crosstalk, TGF-β-driven immune tolerance, lipid mediator class-switching from CysLTs to SPMs, and functional heterogeneity between tissue-resident and inflammatory subsets. Recognizing this functional duality has important implications for interpreting the clinical experience with anti-eosinophil biologics in AR and for designing next-generation precision immunotherapies. From the vantage point of otolaryngology — with its privileged anatomical access to the nasal mucosa — there is a timely opportunity to redefine the role of eosinophils in upper airway allergic disease beyond the classical cytotoxicity narrative.
